# Microwave Assisted Synthesis and Unusual Coupling of Some Novel Pyrido[3,2-f][1,4]thiazepines

**DOI:** 10.3390/molecules16064549

**Published:** 2011-05-31

**Authors:** Rasha M. Faty, Mohamed M. Youssef, Ayman M.S. Youssef

**Affiliations:** 1 Department of Chemistry, Faculty of Science, Cairo University, Egypt; 2 Department of Chemistry, Faculty of Science, Fayoum University, Egypt

**Keywords:** microwave, monothiomalonamide, pyridinecarboxamide, pyridothiazepines, coupling

## Abstract

3-Amino-3-thioxopropanamide (**1**) reacted with ethyl acetoacetate to form 6-hydroxy-4-methyl-2-thioxo-2,3-dihydropyridine-3-carboxamide (**2**), which reacted with α-haloketones **3** to produce 2,3-disubstituted-8-hydroxy-6-methyl-2H,5H-pyrido[3,2-f]-[1,4]thiazepin-5-ones **4a-c**. Benzoylation of **4c** led to the formation of the dibenzoate derivative **9**. Compounds **4a-c** could be prepared stepwise through the formation of S-alkylated derivatives **10a-c**. Compounds **2**, **4a-c**, **9** and **10a-c** were prepared using microwave as a source of heat, and gave better yields in shorter times than those achieved by traditional methods. Coupling of **4a-c** with arenediazonium chlorides proceeded unusually to give the 6-hydroxy-4-methyl-2-(arylazo)thieno[2,3-b]pyridin-3(2H)-one ring contraction products **14**. Structures of the newly synthesized compounds were proven by spectral and chemical methods.

## 1. Introduction

Pyridothiazepines represent a group of less investigated fused heterocyclic compounds. In the last three decades, very few communications have been published regarding the synthesis [1,2,3,4,5,6,7] and biological activities [8,9] of pyridothiazepines. Prompted by these facts, we present here some simple and convenient routes to prepare novel substituted pyrido[3,2-f][1,4]thiazepines by both traditional and microwave assisted methods. 

## 2. Results and Discussion

**Scheme 1:**
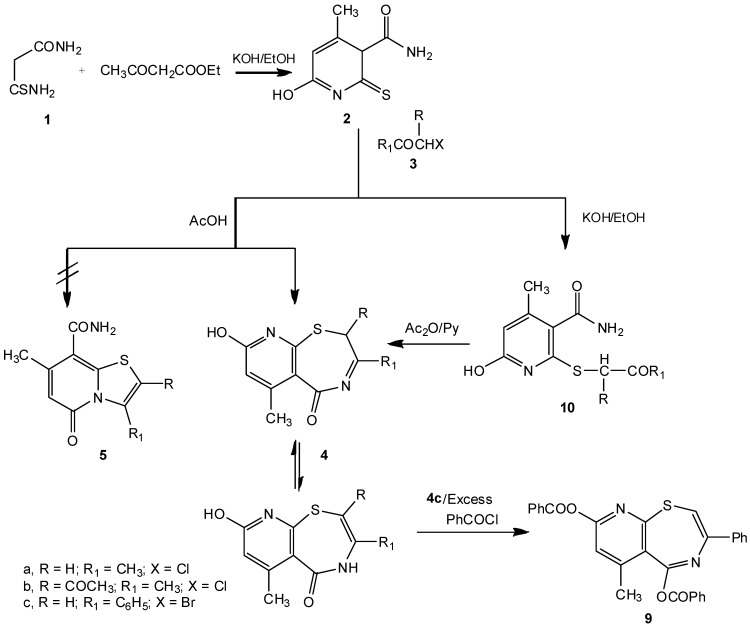
Synthesis and benzoylation of pyrido[3,2-f][1,4]thiazepin-5-ones.

Heating 3-amino-3-thioxopropanamide (monothiomalonamide, **1**) [10] with ethyl acetoacetate in a refluxing ethanolic potassium hydroxide solution led to the formation of 6-hydroxy-4-methyl-2-thioxo-2,3-dihydropyridine-3-carboxamide (**2**) (Scheme 1). 

Compound **2** reacted with α-haloketones **3**, namely chloroacetone (**3a**), 3-chloropentane-2,4-dione (**3b**) and phenacyl bromide (**3c**), in boiling acetic acid to give directly 2,3-disubstituted-8-hydroxy-6-methyl-2*H*,5*H*-pyrido[3,2-f][1,4]thiazepin-5-ones **4a-c** rather than the isomeric thiazolopyridine derivatives **5a-c** (Scheme 1). The preference for structures **4** over **5** was shown by the IR, ^1^H-NMR and ^13^C-NMR spectra. Thus, the IR spectra of compounds **4a,c** displayed only one carbonyl absorption near 1,660 cm^−1^, whereas structure **5** would display two carbonyl absorptions. The ^1^H-NMR and ^13^C-NMR (DMSO-d_6_) of **4c**, as an example, showed a singlet signal at δ 3.66 ppm and a signal at δ 35.3 ppm, respectively, that correspond to z CH_2_ group that would not appear for structure **5c**. Structure **5c** was independently obtained by heating under reflux a mixture of 6-hydroxy-4-methyl-2-thioxo-2,3-dihydropyridine-3-carbonitrile (**6**) [11] with **3c** in aqueous ethanolic potassium hydroxide solution to afford the phenacylthiopyridine-3-carbonitrile derivative **7**, which was converted to **5c** by heating with polyphosphoric acid on a boiling water bath. 

**Scheme 2:**
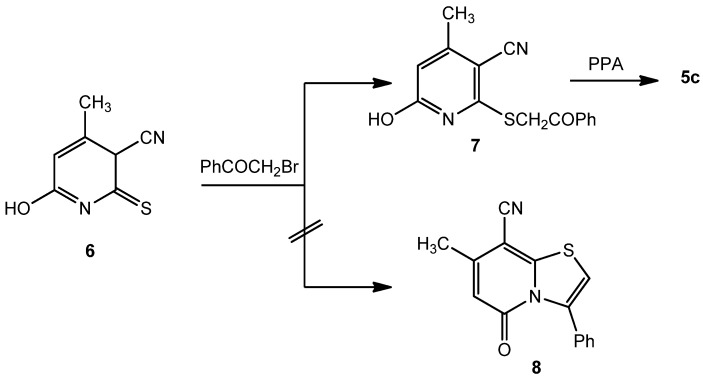
Independent route for the formation of **5c**.

This product was found to be different in all aspects (m.p., mixed m.p. and IR) from the reaction product **4c**, obtained by the reaction between **2** and **3c** in acetic acid. Our original aim was to synthesize the thiazolopyridine-8-carbonitrile **8** by reacting **6** with phenacyl bromide in polyphosphoric acid, and then to hydrolyze product **8** to obtain **5c**. Instead we found that hydrolysis of the carbonitrile group into carboxamide took place directly and **5c** was eventually obtained (Scheme 2). A further confirmation of structure **4** could be obtained by reacting **4c**, as a representative, with an excess of benzoyl chloride in pyridine to obtain the dibenzoate derivative **9**. It is clear that benzoylation is only possible for compound **4c** and not for structure **5**. When **2** was reacted with each of **3a-c** by boiling in ethanolic potassium hydroxide, the S-alkylated derivatives **10a-c** were obtained in poor yields. Compounds **10a-c** could be cyclized by heating with acetic anhydride in pyridine to give **4a-c**, respectively (Scheme 1).

Our research group has recently [12] been interested in performing synthesis of some heterocyclic compounds under the environmentally friendly, time saving microwave-assisted conditions. Accordingly, we re-synthesized the previously described compounds **2**, **4a-c**, **9** and **10a-c** under microwave conditions, aiming to increase reaction yields and reduce the reaction times. The results of these preparation indicated that reaction yields increased by 20–30% compared to the traditional conditions. Reaction times were also significantly reduced. Table 1 summarizes the benefits of using microwave conditions for the synthesis of the above-mentioned compounds.

**Scheme 3:**
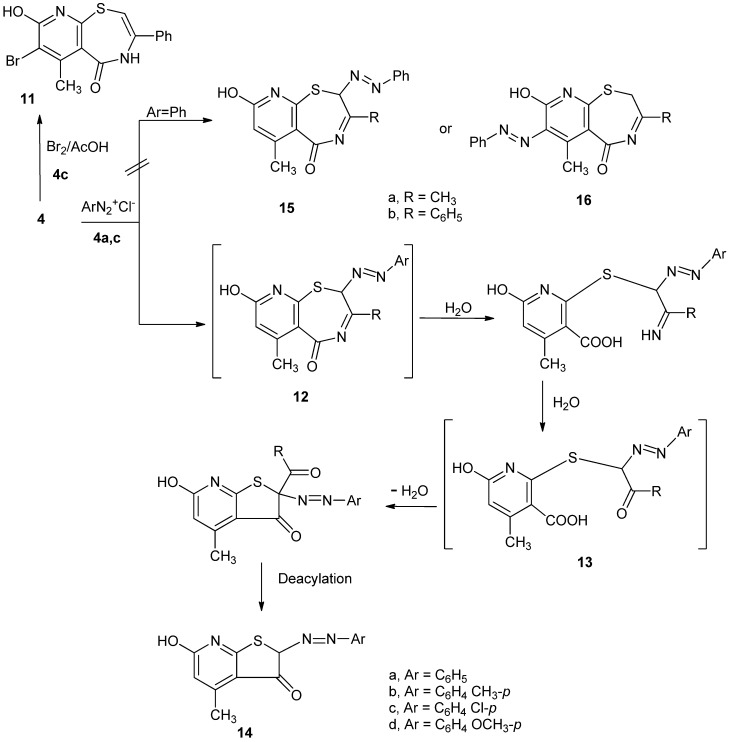
Bromination and explanation of the unusual coupling of **4a,c**.

Compound **4c** underwent bromination upon treatment with bromine in acetic acid to give 7-bromo-6-methyl-3-phenyl-4,5,8,9-tetrahydropyrido[3,2-f][1,4]thiazepine-5,8-dione (**11**) (Scheme 3) The occurrence of bromination at C7 was proved by studying ^1^H-NMR spectrum of **11**, which revealed the disappearance of the signal that originally existed in **4c** at δ6.30 ppm. On the other hand, coupling of **4a-c** with arenediazonium chlorides in pyridine/potassium hydroxide took place in an unexpected manner to produce **14a-c**. 

As an example, treatment of **4a** or **4c** with benzenediazonium chloride gave one and the same reaction product. The mass spectrum and elemental analysis of the reaction product showed that its molecular formula was C_14_H_11_N_3_O_2_S, and could be formulated to be 6-hydroxy-4-methyl-2-(phenylazo)thieno[2,3-b]pyridin-3(2*H*)-one (**14a**). If coupling took place at either C2 or C7 with no further modification, we would have obtained compounds **15a** or **16a**, respectively, each of which has the molecular formula C_16_H_14_N_4_O_2_S or **15b** or **16b**, which would have the molecular formula C_21_H_16_N_4_O_2_S. As a possible sequence for the formation of **14a** from **4a,c**, the latter compounds firstly underwent coupling at position 2 to give the corresponding 2-arylazo derivative, **12**. Compound **12** underwent hydrolysis and gave **13** which underwent cyclization followed by deacylation to give **14a**, as shown in Scheme 3.

**Scheme 4:**
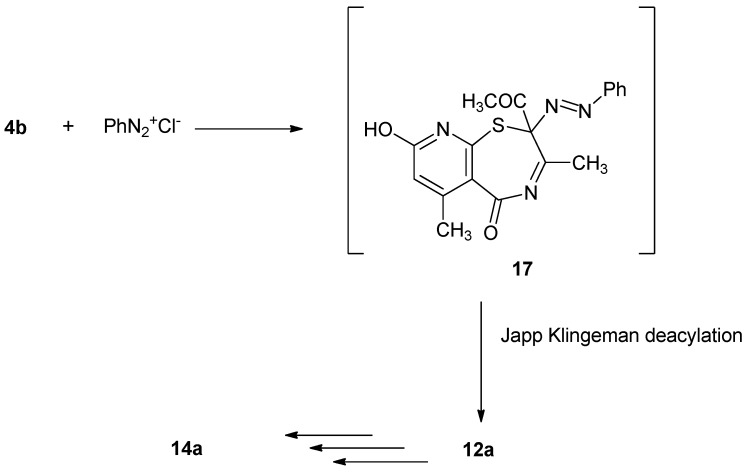
Explanation of formation of **14a** from **4b**.

Surprisingly, coupling of **4b** with benzenediazonium chloride yielded the same reaction product **14a**. The reaction may have proceeded firstly via coupling at position 2 to give the corresponding 2-phenylazo derivative **17** which underwent deacylation (Japp-Klingemann reaction) to give compound **12a**. The latter compound underwent hydrolysis then cyclization followed by deacylation to give **14a** as described in Scheme 4.

## 3. Experimental

### 3.1. General

Melting points were determined in open glass capillaries on a Gallenkamp melting point apparatus and are uncorrected. IR spectra (KBr discs) were recorded on a Shimadzu FTIR-8201PC spectrophotometer. ^1^H-NMR and ^13^C-NMR spectra were recorded on a Varian Mercury 300 MHz and Varian Gemini 200 MHz spectrometers using TMS as an internal standard and DMSO-d_6_ as solvent. Chemical shifts were expressed as δ (ppm) units. Mass spectra were recorded at 70 eV on a Shimadzu GCMS-QP1000EX using an inlet type injector. All reactions were followed by TLC (silica gel, aluminum sheets 60 F254, Merck). The Microanalytical Center of Cairo University performed the microanalyses. Microwave reactions were performed with a Millstone Organic Synthesis Unit (MicroSYNTH with touch control terminal) with a continuous focused microwave power delivery system in a pressure glass vessel (10 mL) sealed with a septum under magnetic stirring. The temperature of the reaction mixture was monitored using a calibrated infrared temperature control under the reaction vessel, and control of the pressure was performed with a pressure sensor connected to the septum of the vessel. 3-Amino-3-thioxopropanamide (monothiomalonamide, **1**) was prepared according to a literature procedure [10].

### 3.2. 6-Hydroxy-4-methyl-2-thioxo-2,3dihydropyridine-3-carboxamide *(**2**)*

*Method A:* A mixture of 3-amino-3-thioxopropanamide (**1**, 1.18 g, 0.01 mol) and ethyl acetoacetate (1.30 g, 0.01 mol) was heated under reflux in ethanolic potassium hydroxide solution (0.56 g, 0.01 mole in 20 mL ethanol) for 3 h. The formed white potassium salt was dissolved in water and acidified with hydrochloric acid. The precipitated solid was filtered off, washed with ethanol, dried and recrystallized from ethanol.

*Method B:* The same reactants of method A were heated in the microwave apparatus at 500 w and 140 °C for 15 min. The reaction mixture was treated in a similar manner to method A to afford compound **2**. Compound **2** was obtained as white crystals in 55% yield (Method A) and 82% yield (Method B), m.p. 194–195 °C. ^1^H-NMR: 2.30 (s, 3H, CH_3_), 3.85 (br s, 1H, OH, D_2_O exchangeable), 5.24 (s, 1H, H-3), 6.10 (s, 1H, H-5), 7.40 (br s, 2H, NH_2_, D_2_O exchangeable); ^13^C-NMR: 20.3 (CH_3_), 71.5 (C-3), 112.1, 137.3, 162.3 (sp2 C), 173.1 (CO), 188.0 (CS); IRν: 3307, 3175 (NH, OH) and 1648 (CO); MS: M^+^ *m*/*z* 184.0 (21.1%); Anal. Calcd. for C_7_H_8_N_2_O_2_S (184.22): C (45.64%), H (4.38%), N (15.21%), S (17.41%). Found: C (45.70%), H (4.20%), N (15.10%), S (17.30%).

### 3.3. 2,3-disubstituted-8-hydroxy-6-methyl-2H,5H-pyrido[3,2-f][1,4]thiazepin-5-ones ***4a-c***

*Method A:* A mixture of **2** (1.84 g, 0.01 mole) and α-haloketones **3a-c** (0.01 mole) was heated under reflux in glacial acetic acid (30 mL) for 10 hours. The reaction mixture was then poured onto water; the deposited precipitate was filtered off, dried and crystallized from the proper solvent.

*Method B:* The same reactants of method A were heated in microwave at 500 w and 140 °C for 30 min. The reaction mixture was treated in a similar manner to method A to give compounds **4a-c**.

*3,6-Dimethyl-8-hydroxy-2H,5H-pyrido[3,2-f]*[1,4]*thiazepin-5-one* (**4a**). Crystallized from ethanol as white crystals in 61% yield (Method A) and 85% yield (Method B); m.p. 286–287 °C. ^1^H-NMR: 2.36 (s, 3H, CH_3_), 2.49 (s, 3H, CH_3_), 3.66 (s, 2H, CH_2_), 6.23 (s, 1H, pyridine-H), 12.42 (s, 1H, OH, D2O exchangeable). ^13^C-NMR: 20.1 (CH_3_), 21.8 (CH_3_), 34.7 (CH_2_), 114.1, 116.9, 145.2, 155.1, 161.2, 165.3 (sp2 C), 179.8 (CO). IRν: 3430 (OH) and 1655 (CO). MS: M^+^ *m*/*z*: 222 (5.7%); Anal. Calcd for C_10_H_10_N_2_O_2_S (222.26): C (54.05%), H (4.53%), N (12.60%), S (14.43%). Found: C (54.00%), H (4.30%), N (12.50%), S (14.60%). 

*(3,6-Dimethyl-4,5-dihydro-8-hydroxy-5-oxopyrido[3,2-f]*[1,4]*thiazepin-2-yl)ethanone* (**4b**). Crystallized from acetic acid as white crystals in 59% yield (Method A) and 80% yield (Method B); m.p. 270–271 °C. ^1^H-NMR: 2.36 (s, 3H, CH_3_), 2.46 (s, 3H, CH_3_), 2.54 ppm (s, 3H, CH_3_), 6.22 (s, 1H, pyridine-H), 12.45 (br.s, 2H, OH, NH, D_2_O exchangeable); ^13^C-NMR: 20.3 (CH_3_), 20.8 (CH_3_), 28.1 (CH_3_), 59.0 (C-2), 114.9, 117.0, 145.6, 155.4, 163.2, 165.6 (sp2 C); IRν: 3429 (NH) and 1732 & 1659 (2 CO). MS: M^+^ *m*/*z*: 264 (1.3%); Anal. Calcd for C_12_H_12_N_2_O_3_S (264.30): C (54.53%), H (4.58%), N (10.60%), S (12.13%). Found: C (54.60%), H (4.30%), N (10.50%), S (12.20%). 

*8-Hydroxy-6-methyl-3-phenyl-2H,5H-pyrido[3,2-f]*[1,4]*thiazepin-5-one* (**4c**). Crystallized from acetic acid as yellowish white crystals in 56% yield (Method A) and 85% yield (Method B); m.p. 330–331 °C. ^1^H-NMR: 2.55 (s, 3H, CH_3_), 3.50 (s, 2H, CH_2_), 6.30 (s, 1H, pyridine-H), 7.65-7.80 (m, 3H, Ar-H), 7.90–8.00 (m, 2H, Ar-H), 12.65 (br s, 1H, OH, D_2_O exchangeable). ^13^C-NMR: 20.1 (CH_3_), 35.3 (CH_2_), 114.1, 117.2, 128.3, 129.0, 132.1, 134.5, 145.2, 155.3, 162.7, 165.2 (sp2 C), 180.3 (CO); IRν: 3440 (broad, OH), 2924 (CH) and 1690 (CO). MS: M^+^ *m*/*z*: 284 (100%); Anal. Calcd for C_15_H_12_N_2_O_2_S (284.33): C (63.36%), H (4.25%), N (9.85%), S (11.28%); Found: C (63.10%), H (4.40%), N (10.00%), S (11.40%).

### 3.4. 6-Hydroxy-4-methyl-2-((2-oxo-2-phenylethyl)thio)pyridine-3-carbonitrile *(**7**)*

Compound **6** (1.66 g, 0.01 mol, [11]) was dissolved in a warm ethanolic potassium hydroxide solution [prepared by dissolving 0.56 g (0.01 mol) of potassium hydroxide in 50 mL of ethanol]. Then **3c** (1.99 g, 0.01 mol) was added. The mixture was refluxed for two hours, then cooled and poured into water. The solid so-formed was filtered off and recrystallized from dilute dimethylformamide to obtain white crystals in 73% yield, m.p. 211–212 °C. ^1^H-NMR: 2.43 (s, 3H, CH_3_), 4.70 (s, 2H, CH_2_), 6,11 (s, 1H, pyridine-H), 7.55–7.94 (m, 5H, Ar-H), 12.13 (s, 1H, OH, D_2_O exchangeable); ^13^C-NMR: 21.2 (CH_3_), 38.7 (CH_2_), 116.1 (CN), 117.0, 117.8, 128.6, 129.3, 133.1, 135.7, 152.2, 156.6, 159.0 (sp2 C), 190.3 (CO); IRν: 2980–3215 (broad, OH), 2180 (CN), 1710 (CO); MS: M^+^ *m*/*z*: 284 (15%). Anal. Calcd. for C_15_H_12_N_2_O_2_S (284.33): C (63.36%), H (4.25%), N (9.85%), S (11.28%). Found: C (63.10%), H (4.20%), N (9.70%), S (11.40%).

### 3.5. 7-methyl-5-oxo-3-phenyl-5H-thiazolo[3,2-a]pyridine-8-carboxamide *(**5c**)*

Compound **7** (1 g) was heated in polyphosphoric acid (3 g phosphorus pentoxide in 15 mL ortho- phosphoric acid) at 140 °C for three hours. Then the reaction mixture was allowed to cool and poured into water. The solid product so-precipitated was filtered off, washed with water and recrystallized from dimethylformamide to form **5c**, obtained as white crystals in 60% yield, m.p. 349–350 °C. ^1^H-NMR: 2.13 (s, 3H, CH_3_), 6.15 (s, 1H, pyridine-H), 6.35 (s, 1H, thiazolo-H), 7.30–7.55 (m, 5H, Ar-H), 7.70 (s, 2H, NH_2_, D_2_O, exchangeable); ^13^C-NMR: 21.3 (CH_3_), 106.0, 118.4, 127.3, 128.5, 128.9, 135.4, 138.1, 145.1, 146.0 (sp2 C), 158.8 (CO), 173.0 (CO); IRν: 3422, 3131 (NH_2_), 1673, 1650 (2 CO); MS: M^+^ *m*/*z*: 284 (55%). Anal. Calcd. for C_15_H_12_N_2_O_2_S (284.34): C (63.36%), H (4.25%), N (9.85%), S (11.28%). Found: C (63.50%), H (4.40%), N (10.0%), S (11.40%).

### 3.6. 6-Methyl-3-phenylpyrido[3,2-f][1,4]thiazepine-5,8-dibenzoate *(**9**)*

*Method A:* To a solution of **4c** (2.84 g, 0.01 mol) in pyridine (50 mL), benzoyl chloride (0.02 mol) was added drop-wise with stirring at room temperature. The mixture was then refluxed for three hours and then diluted with cold water (30 mL). The resultant crude product thus precipitated was collected by filtration, washed with water, dried and crystallized from dioxane to afford **9**. 

*Method B:* The same reactants of method A were heated in microwave at 500 w and 140 °C for 15 min. The reaction mixture was treated in a similar manner to method A to obtain compound **9**. Compound **9** was obtained as white crystals in 57% yield (Method A) and 79% yield (Method B), m.p. 150–151 °C. ^1^H-NMR: 2.30 (s, 3H, CH_3_), 6.25 (s, 1H, pyridine-H), 7.30–8.20 (m, 16H, Ar-H); ^13^C-NMR: 19.8 (CH_3_), 106.3, 118.3, 119.4, 126.7, 127.1, 128.8, 130.3, 133.8, 134.9, 137.1, 145.0, 155.7, 158.3, 160.0 (sp2 C), 163.4 (CO), 166.0 (CO). IRν: 3144 (CH), 2950 (CH), 1739 (CO). MS: M^+^ *m*/*z*: 492 (2%). Anal. Calcd for C_29_H_20_N_2_O_4_S (492.55): C (70.72%), H (4.09%), N (5.69%), S (6.51%). Found: C (70.80%), H (4.30%), N (5.80%), S (6.70%)

### 3.7. 2-[(1,2-Disubstituted)oxoethylthio]-6-hydroxy-4-methylpyridine-3-carboxamides ***10a-c***

*Method A:* 1.84 g (0.01 mol) of **2** was dissolved in a warm ethanolic potassium hydroxide solution [prepared by dissolving 0.56 g (0.01 mol) of potassium hydroxide in 50 mL of ethanol]. Then the proper α-haloketone (0.01 mol) was added. The mixture refluxed for 10 minutes; the mixture then stirred at room temperature overnight, then cooled and poured into water. The formed solid product was filtered off and crystallized from ethanol.

*Method B:* The same reactants of method A were heated in microwave at 500 w and 90 °C for 5 min. The reaction mixture was treated in a similar manner to method A to obtain compounds **10a-c**.

*2-[6-Hydroxy-4-methyl-2-((2-oxopropyl)thio)]pyridine-3-carboxamide* (**10a**). Obtained as white crystals in 25% yield (Method A) and 55% (Method B); m.p. 241–242 °C. ^1^H-NMR: 2.00 (s, 3H, CH_3_), 2.50 (s, 3H, CH_3_), 4.30 (s, 2H, CH_2_), 6.15 (pyridine-H), 7.65 (s, 2H, NH_2_, D_2_O exchangeable), 11.75 (s, 1H, OH, D_2_O exchangeable); ^13^C-NMR: 19.3 (CH_3_), 28.1 (CH_3_), 48.3 (CH_2_), 112.1, 116.2, 146.7, 155.2, 156.0 (sp2 C), 168.4 (CO), 179.3 (CO); IRν: 3295–3446 (broad, NH, OH), 1732, 1678 (2 CO); MS: M^+^ *m*/*z*: 240 (37%). Anal. Calcd for C_10_H_12_N_2_O_3_S (240.28): C (49.99%), H (5.03%), N (11.66%), S (13.34%). Found: C (50.10%), H (4.90%), N (11.30%), S (13.60%).

*2-(2,4-Dioxopentan-3-ylthio)-6-hydroxy-4-methylpyridine-3-carboxamide* (**10b**). Obtained as white crystals in 31% yield (Method A) and 45% yield (Method B); m.p. 311–312 °C. ^1^H-NMR: 2.20 (s, 3H, CH_3_), 2.65 (s, 6H, 2CH_3_), 4.80 (s, 1H, CH), 6.20 (s, 1H, pyridine-H), 7.60 (s, 2H, NH_2_, D_2_O exchangeable), 12.00 (s, 1H, OH, D_2_O exchangeable); ^13^C-NMR: 19.1 (CH_3_), 28.5 (CH_3_), 78.5 (CH), 111.4, 116.8, 146.1, 155.5, 156.4 (sp2 C), 168.0 (CO), 185.3 (CO); IRν: 3288–3433 (broad, NH, OH), 1705, 1678 (2 CO). MS: M^+^ *m*/*z*: 282 (12%). Anal. Calcd for C_12_H_14_N_2_O_4_S (282.32): C (51.05%), H (5.00%), N (9.92%), S (11.36%). Found: C (51.20%), H (4.90%), N (10.00%), S (11.00%).

*2-[6-Hydroxy-4-methyl-2-((2-oxo-2-phenylethyl)thio)]pyridine-3-carboxamide* (**10c**). Obtained as white crystals in 41% yield (Method A) and 63% yield (Method B); m.p. 276–277 °C. ^1^H-NMR: 2.10 (s, 3H, CH_3_), 4.95 (s, 2H, CH_2_), 6.25 (s, 1H, pyridine-H), 7.30 (s, 2H, NH_2_, D_2_O exchangeable), 7.56–7.94 (m, 5H, Ar-H), 11.95 (s, 1H, OH, D_2_O exchangeable); ^13^C-NMR: 20.1 (CH_3_), 38.5 (CH_2_), 112.5, 116.1, 128.5, 128.9, 134.2, 136.0, 146.4, 155.2, 156.8 (sp2 C), 168.3 (CO), 189.0 (CO); IRν: 3471(NH, OH), 1712, 1666 (2 CO). MS: M^+^ *m*/*z*: 302 (21.6%). Anal. Calcd for C_15_H_14_N_2_O_3_S (302.35): C (59.59%), H (4.67%), N (9.27%), S (10.61%). Found: C (59.30%), H (4.30%), N (9.30%), S (10.60%).

### 3.8. 7-Bromo-6-methyl-3-phenylpyrido[3,2-f][1,4]thiazepine-5,8(4H,9H)-dione *(**11**)*

To a solution of **4c** (2.84 g, 0.01 mol) in glacial acetic acid (50 mL), bromine (0.5 mL, 0.01 mole) was added dropwise with stirring at room temperature under sunlight. The mixture was then stirred at water bath for two hours and then diluted with cold water (30 mL). The resultant crude product thus precipitated was collected by filtration, washed with water, dried and crystallized from dilute dimethylformamide to afford **11** as white crystals in 73% yield; m.p. 310–311 °C. ^1^H-NMR: δ 2.58 (s, 3H, CH_3_), 7.60–7.86 (m, 6H, Ar-H + thiazepine-H), 13.40 (bs, 2H, NH+OH, D_2_O exchangeable). ^13^C-NMR: 12.3 (CH_3_), 100.2, 108.0, 111.3, 126.9, 127.8, 134.5, 135.6, 137.7, 142.0, 148.4, 152.1 (sp2 C), 165.6 (CO); IRν: 3180-3341 (broad, NH, OH), 1678, (CO). MS: M^+^ *m*/*z*: 365 (64%), 363.0 (66%). Anal. Calcd for C_15_H_11_BrN_2_O_2_S (363.97): C (49.60%), H (3.05%), Br (22.00%), N (7.71%), S (8.83%). Found: C (50.00%), H (3.30%), Br (21.70%), N (7.52%), S (8.60%).

### 3.9. 3-Hydroxy-4-methyl-2-(p-substituted phenylazo)-6,7-dihydrothieno[2,3-b]pyridine-6-ones ***14a-c***

*General procedure:* To a cold solution of **4** (0.01 mole) in pyridine [50 mL, containing potassium hydroxide, 0.3 g], the arenediazonium chloride (0.01 mL) [prepared by adding concentrated hydrochloric acid (3 mL) to aromatic amine (0.01 mole) at 0 °C and treating the resulting hydrochloride with a cold solution of sodium nitrite 0.69 g (0.01 mole) in water (5 mL)] was added drop-wise with stirring at 0 °C. The coupling mixture was stirred at room temperature for two hours and then diluted with water (30 mL). The crude product thus precipitated was collected by filtration, washed with water, dried and crystallized from dimethylformamide.

*3-Hydroxy-4-methyl-2-(phenylazo)-6,7-dihydrothieno[2,3-b]pyridine-6-one* (**14a**). Obtained in 83% yield as an orange crystals; m.p. 320–321 °C. ^1^H-NMR: 2.44 (s, 3H, CH_3_), 3.45 (s, 1H, thiazoline-H), 6.20 (s, 1H, pyridine-H), 7.00–7.45 (m, 5H, Ar-H), 11.89 (s, 1H, OH, D_2_O exchangeable); ^13^C-NMR: 19.3 (CH_3_), 75.5 (thiazoline C), 113.5, 115.5, 122.3, 126.4, 129.0, 144.3, 151.1, 155.7, 157.3 (sp2 C), 188.2 (CO); IRν: 3210–2927 (broad, OH), 1639 (CO). MS: M^+^*m*/*z*: 285 (7.3%). Anal. Calcd for C_14_H_11_N_3_O_2_S (285.32): C (58.93%), H (3.89%), N (14.73%), S (11.24%). Found: C (58.90%), H (4.00%), N (14.50%), S (11.60%).

*3-Hydroxy-4-methyl-2-(p-tolylazo)-6,7-dihydrothieno[2,3-b]pyridine-6-one* (**14b**). Obtained in 91% yield as orange crystals; m.p. 338–339 °C. ^1^H-NMR: δ 2.27 (s, 3H, CH_3_), 2.43 (s, 3H, CH_3_), 3.40 (s, 1H, thiazoline-H), 6.14 (s, 1H, pyridine-H), 7.14 (d, 2H, aromatic protons), 7.23 (d, 2H, aromatic protons), 11.61 (s, 1H, OH, D_2_O exchangeable). ^13^C-NMR: 18.9 (CH_3_), 22.3 (CH_3_), 74.8 (thiazoline c), 113.8, 115.6, 122.9, 130.1, 135.4, 144.8, 147.7, 155.2, 156.6 (sp2 C), 189.0 (CO); IRν: 3200–2900 (broad, OH), 1651 (CO). MS: M^+^ *m*/*z*: 299 (100%). Anal. Calcd for C_15_H_13_N_3_O_2_S (299.35): C (60.18%), H (4.38%), N (14.04%), S (10.71%). Found: C (60.30%), H (4.30%), N (14.20%), S (10.60%). 

*3-Hydroxy-4-methyl-2-(p-chlorophenylazo)-6,7-dihydrothieno[2,3-b]pyridine-6-one* (**14c**). Obtained in 85% yield as orange crystals; m.p. 340–341 °C. ^1^H NMR: δ 2.44 (s, 3H, CH_3_), 3.45 (s, 1H, thiazoline-H), 6.13 (s, 1H, pyridine-H), 7.27–7.39 (m, 4H, aromatic-H), 13.40 (s, 1H, OH, D_2_O exchangeable). ^13^C-NMR: 18.4 (CH_3_), 73.5 (thiazoline C), 114.3, 115.8, 121.9, 130.8, 135.9, 145.8, 149.7, 155.7, 156.9 (sp2 C), 190.4 (CO); IRν: 3217–2936 (broad, OH), 1651 (CO). MS: M^+^ *m*/*z*: 319 (100%). Anal. Calcd for C_14_H_10_ClN_3_O_2_S (319.77): C (52.59%), H (3.15%), Cl (11.09%), N (13.14%), S (10.03%). Found: C (52.76%), H (3.30%), Cl (11.40%), N (13.30%), S (10.30%).

*3-Hydroxy-4-methyl-2-[(p-methoxyphenyl)azo]-6,7-dihydrothieno[2,3-b]pyridine-6-one* (**14d**). Obtained in 83% yield as orange crystals; m.p. 316–317 °C. ^1^H-NMR: δ 2.45 (s, 3H, CH_3_), 3.55 (s, 1H, thiazoline-H), 3.74 (s, 3H, OCH_3_), 6.13 (s, 1H, pyridine-H), 6.94 (d, 2H, aromatic protons), 7.25 (d, 2H, aromatic protons), 12.84 (s, 1H, OH, D_2_O exchangeable). ^13^C-NMR: 18.8 (CH_3_), 55.6 (OCH_3_), 73.5 (thiazoline C), 114.1, 115.0, 115.9, 123.8, 143.9, 145.0, 154.7, 156.7, 157.4 (sp2 C), 188.4 (CO); IRν: 3433–2943 (broad, OH), 1655 (CO). MS: M^+^ *m*/*z*: 315 (100%). Anal. Calcd for C_15_H_13_N_3_O_3_S (315.35): C (57.13%), H (4.16%), N (13.33%), S (10.17%). Found: C (56.90%), H (4.40%), N (13.20%), S (10.10%).

## 4. Conclusions

Several new pyridothiazepines have been synthesized using both traditional methods and microwave assisted conditions. The latter methods proved much more efficient in reducing reaction times as well as increasing the overall yield of the reactions. Structures of the newly synthesized compounds were proven by both spectral and chemical methods.

## Figures and Tables

**Table 1:** Comparison between traditional methods and microwave assisted methods of synthesis of compounds **2**, **4**, **9** and **10**.

Compound no.	Reaction Time		Reaction Yield %	
	Traditional Method	Microwave	Traditional Method	Microwave
**2**	3h	15 min.	55	82
**4a**	10h	30 min.	61	85
**4b**	10h	30 min.	59	80
**4c**	10h	30 min.	56	85
**9**	3h	15 min.	57	79
**10a**	10 min. reflux + overnight at r.t.	5 min.	25	55
**10b**	10 min. reflux + overnight at r.t.	5 min.	31	45
**10c**	10 min. reflux + overnight at r.t.	5 min.	41	63
